# Availability and readiness of public health facilities to provide differentiated service delivery models for HIV treatment in Zambia: implications for better treatment outcomes

**DOI:** 10.3389/fpubh.2024.1396590

**Published:** 2024-11-06

**Authors:** Patrick Kaonga, Mutale Sampa, Mwiche Musukuma, Mulanda Joseph Mulawa, Mataanana Mulavu, Doreen Sitali, Given Moonga, Oliver Mweemba, Tulani Francis Matenga, Cosmas Zyambo, Twaambo Hamoonga, Henry Phiri, Hikabasa Halwindi, Malizgani Paul Chavula, Joseph Mumba Zulu, Choolwe Jacobs

**Affiliations:** ^1^Department of Epidemiology and Biostatistics, School of Public Health, University of Zambia, Lusaka, Zambia; ^2^Department of Health Promotion and Education, School of Public Health, University of Zambia, Lusaka, Zambia; ^3^Department of Community and Family Medicine, School of Public Health, University of Zambia, Lusaka, Zambia; ^4^Department of Population Studies and Global Health, School of Public Health, University of Zambia, Lusaka, Zambia; ^5^The Global Fund Unit, Ministry of Health, Lusaka, Zambia; ^6^Department of Health Policy and Management, School of Public Health, University of Zambia, Lusaka, Zambia

**Keywords:** HIV, differentiated service delivery, availability, readiness, Zambia

## Abstract

**Background:**

There is persistent pressure on countries with a high burden of HIV infection to reach desired targets for HIV treatment outcomes. This has led to moving from the “one-size-fits-all” model to differentiated service delivery (DSD) models, which are meant to be more patient-centered and efficient but without compromising on the quality of patient care. However, for DSD models to be efficient, facilities should have indicators of HIV services available and ready to provide the DSD models. We aimed to assess the availability of HIV service indicators and the readiness of facilities to provide DSD models for HIV treatment in selected public health facilities in Zambia.

**Methods:**

We conducted a nationwide cross-sectional survey among public health facilities in Zambia that provide antiretroviral therapy (ART) services. We used an interviewer-administered questionnaire based on a World Health Organization (WHO) Service Availability Readiness Assessment (SARA) tool to assess the availability of HIV service indicators and the readiness of facilities to implement DSD models for HIV treatment. Availability and readiness were considered latent constructs, and therefore, we used structural equation modeling (SEM) to determine the correlations between them and their respective indicators.

**Results:**

Of 60 public health ART facilities, the overall availability of HIV service indicators was 80.0% (48/60), and readiness to provide the DSD models was 81.7% (48/60). However, only 48 and 39% of the facilities had all indicators of availability and readiness, respectively. Retention in care for HIV multidisciplinary teams was more likely to occur in urban areas than in rural areas. SEM showed that the standardized estimate between availability and readiness was significantly and positively correlated (*r* = 0.73, *p* < 0.0001). In addition, both availability and readiness were significantly and positively correlated with most of their respective indicators.

**Conclusion:**

Although most facilities had available HIV service indicators and were ready to provide DSD models, most facilities did not have all indicators of availability and readiness. In addition, there were differences between rural and urban facilities in some indicators. There is a need for persistent and heightened efforts meant to implement DSD in HIV treatment, especially in rural areas to accelerate reaching the desired HIV treatment outcomes.

## Introduction

There has been mounting pressure to meet global targets for HIV treatment, such as the 95-95-95 goals, while also reducing unnecessary burdens and costs for both patients and the healthcare system. In addition, there is a need to better titrate limited healthcare system resources to diverse client needs and improved treatment outcomes, better retention in care, increased peer support, higher viral load suppression, reduced waiting time and clinic visits, and extended time for ART refill (1, 2). This requires a shift away from the traditional “one-size–fits-all” model to alternative approaches such as HIV differentiated service delivery (DSD) models that are more patient-centered and efficient while not compromising patient care (3). DSD models are meant to simplify HIV treatment services across the HIV cascade to better reflect the preferences, expectations, and needs of people living with HIV (4, 5). As almost every country in sub-Saharan Africa is scaling up the DSD models, with a decline in funding, various funders, policymakers, and governments are questioning which models are more efficient and effective for greater client treatment uptake and coverage and better HIV treatment outcomes (6, 7). The DSD models offer an exciting and promising alternative to HIV treatment, but it is unclear which models are most effective and relevant given the diverse settings and populations. To maximize the effect and impact of DSD models, healthcare workers need to acknowledge and appreciate patient-centered and adaptive approaches and employ quality improvement processes. However, routine implementation of DSD models, in most instances, does not align with monitoring and evaluation strategies needed to assess the impact of DSD models on key HIV treatment outcomes. The pressure to roll out DSD models, especially in public health facilities, poses valid questions bordering on the availability of HIV service indicators and the readiness of health facilities to provide DSD models.

In 2017, Zambia adopted the DSD strategy, and the models recommended in the country for stable clients (clients with no adverse drug reactions that require regular monitoring, no current illnesses, and evidence of treatment success: two consecutive viral load measurements of <1,000 copies/mL and a CD4 count of 200 cells/mm^3^) (8) are:

- Multi-month dispensing: Clients are given ART from a health facility lasting for several months.
- Fast track: Clients are given ART from a facility or dispensing point without being attended to by a clinician or it could be from a separate queue or kiosk, at a facility to speed up service delivery.
- After hours: It denotes additional hours to a facility’s operations to facilitate access for clients who cannot manage normal working hours, such as on evenings or weekends.
- Home delivery: This refers to delivering ART to patients’ homes (e.g., by a community health worker). Community delivery ART points to a variety of models that bring both clinical care and medications into the community, such as nurse-led outreach.
- Scholar: This is a model for those in schools or learning institutions where ART pickup and other appointments are provided during the holidays, weekends, or after hours.
- Dedicated pediatric ART day: Dedicated day for pediatrics clinics and medication pickups timed to coincide with school holidays.
- Adolescent support: This is a facility-based model for adolescents and young women/men aged 10–24 years. The model aims to ensure uninterrupted, age-appropriate, and comprehensive care before, during, and after the transition to adult care.
- Men’s clinic: A facility-based model for men aged 15 years and older, featuring a separate space away from the main clinic. This model is combined with various integrated services such as prostate screening, male circumcision, erectile dysfunction, and condom distribution (1).

Empirical evidence suggests that core healthcare services, such as infrastructure, key health personnel, and service utilization, along with facility readiness components, including medicines, standard precautions, basic equipment, laboratory tests, and commodities, are essential prerequisites for optimal healthcare delivery. Assessment of these indicators is more feasible and cost-effective than downstream outcome indicators of service quality (9, 10). The World Health Organization (WHO) recommends the use of the Service Availability and Readiness Assessment (SARA) tool to assess healthcare service availability and readiness. Utilization of this tool can inform planners and implementers of health programs in terms of human resources, supplies, and essential services (6).

Both availability and readiness have many facets and are latent constructs. Therefore, the use of structural equation modeling (SEM) could provide a more reliable measure of the relationship between indicators of availability and readiness with these latent constructs. We hypothesized that there is a relationship between availability and readiness since the availability of the required HIV service indicators at a facility could suggest readiness and vice versa. The premise is that the absence of components of availability or readiness would negatively affect the implementation and later adoption of the DSD models.

In Zambia, as far as we searched the literature, we did not come across any study that assessed the availability of HIV service indicators and readiness to provide DSD models in public health facilities. We anticipated differences between rural and urban facilities rather than by type of facility based on the implementation and scaling-up of the DSD models (1). Therefore, this study set out to assess the availability of HIV service indicators and the readiness of public health facilities to provide DSD models. This was performed to identify gaps in the current implementation strategies as the first step toward strengthening indicators and tracking the availability of HIV service indicators and the readiness of facilities to effectively provide differentiated service delivery for HIV treatment and adoption.

## Methods

### Study setting and design

Zambia is a landlocked, lower-middle-income country located in Southern Africa with an estimated population of 20 million people. Most (60%) of the population reside in rural areas, and the country is divided into 10 provinces. Approximately half (50%) of the population is under the age of 15 years, and only 3% are above the age of 65 years (11). The country has an HIV prevalence of 11% among adults (15+ years), and women are disproportionately affected, with a 1.7 times higher prevalence than men (12).

The government of Zambia through the Ministry of Health together with implementing partners launched its first DSD models as a pilot in 2016 and began scaling up in 2017 (1). DSD models are implemented with an emphasis on increasing community DSD coverage following the rollout of updated consolidated HIV guidelines. The President’s Emergency Plan for AIDS Relief (PEPFAR) and the Ministry of Health together with other partners aim to provide and scale HIV services to achieve HIV epidemic control. In this regard, Zambia has made tremendous progress, where 88.7% of adults (15+ years) living with HIV are aware of their HIV status; among adults living with HIV who are aware of their status, 98.0% are on ART, and among adults who are on ART, 96.3% have viral load suppression (1). The country has over 1996 ART facilities, which are supported by PEPFAR to provide HIV services through several implementing partners.

This was a cross-sectional survey conducted in 10 selected districts across 8 provinces, namely, Solwezi, Ndola, Kabwe, Mansa, Choma, Livingstone, Kapiri Mposhi, Luangwa, Chipata, and Lusaka districts.

### Recruitment of study sites

This study included public health facilities providing HIV treatment. In Zambia, the delivery system of the healthcare service has three levels: (i) First level: community-level health facilities including district hospitals, health centers, and health posts; (ii) second-level: provincial or general hospitals; and (ii) third level: central or specialist hospitals. This study included facilities in the first level (health posts, health centers, level 1 hospitals, and level 2 hospitals) serving both rural and urban populations. We included facilities that were providing HIV prevention, testing, and treatment services. There were major differences in terms of patients’ volume, facility organization, and staffing levels. These differences may affect patients’ acceptability of DSD models. According to the Ministry of Health records, there were 1992 public ART facilities at the time of the study. We excluded low-volume facilities (<500 clients on ART), facilities with less than three DSD models implemented, and those with less than 12 months of experience providing ART and DSD models.

### Sample size

We adopted a multi-stage cluster sampling. Our initial stage involved the random selection of 10 districts in 8 provinces. We anticipated a minimum sample size of five facilities in each district with an expected deviation of <4, level of confidence = 95%, precision = 1, inter-class correction coefficient of <0.02, power = 80%, and cluster size of 4 (13). We increased the number of facilities to 20 per district based on the information from the Ministry of Health (approximately 25% would be high-volume facilities with >500 clients and at least 3 DSD models implemented), which would meet the inclusion criteria. Based on this information, the number of facilities increased from 5 to 6. Then, 60 facilities were randomly chosen in the second stage using probability proportional to size.

### Measurement of variables

In this study, there were two latent constructs as outcome variables, namely, availability and readiness based on the WHO recommendations for assessing facility services availability and readiness. Therefore, the availability of HIV service indicators was considered as the physical presence of the delivery of HIV services (14), while readiness is the capacity of health facilities to provide specific healthcare services (15); in this case, it was DSD models for HIV treatment. Both constructs can be measured through tracer items such as guidelines, trained staff, and commodities (16). In this study, the availability of HIV service indicators was measured by the following nine items: “an existing HIV multidisciplinary team”, “latest ART orientation guidelines rolled out”, “adequate storage space for additional commodities related to HIV services”, “implemented the Zambian HIV quality improvement framework”, “achieved a routine viral load monitoring uptake of ≥90%”, “established a facility-based system for fast-track ART distribution”, “had ≥ 3 months of ART available on site”, “established system to monitor patients level outcomes specifically retention, lost to follow-up, mortalities and viral load suppression”, and “established recording and reporting systems for community ART.” Readiness was measured using the following indicators: “presence of community health workers in all departments offering HIV services oriented on the latest ART guidelines for the year 2022”, “had a commodity management or commodity security committee”, “does the facility have a quality improvement team”, “had an HIV multidisciplinary team to review clinical cases and provide support to patients failing HIV treatment or with advanced disease”, “healthcare workers trained on the revised HIV monitoring and evaluation tools”, “identified a focal person to oversee community-based ART distribution”, “identified appropriate personnel to distribute ART”, “had staff and resources to train ART distributors”, and “identified a focal person to pre-pack and label ART for community distribution”. The outcome variables were then created as composite scores by adding the presence of each indicator (present = 1; not present = 0), and for both, if all indicators were present, a maximum score of nine was given and if all indicators were not present, a score of zero was given. Facilities with 50% or more available HIV service indicators and readiness were categorized as available and ready, respectively.

### Data collection procedure

Data were collected using KOBO Collect (17), a mobile platform based on the WHO health facility SARA assessment tool. All the questions contained in the tool regarding service availability and readiness had Yes/No responses. Data on both availability and readiness were collected from the ART department of the facilities. In addition, we obtained information regarding the number of clients per facility, average waiting time for clients to be seen at the ART clinic, whether the facility is rural or urban, ART operational times, average distance clients cover to get to the facility, ART regimen dispensed, and types of DSD models implemented at the facility.

### Development and validation of the availability and readiness tool

Briefly, we conducted a literature search for availability of HIV service indicators and readiness to implement DSD models instrument, no prior validated tool was found. Thus, local Zambian experts in HIV DSD models and HIV services were engaged in brainstorming session to evaluate whether the questions effectively captured availability and readiness of facilities to implement DSD models. This was followed by a psychometrician checking for any errors and later was piloted in five facilities and assessed face validity of the questions to confirm clarity and the meaning of the questions. Data from these facilities was not included in the final analysis. Internal consistency was assessed using Cronbach Alpha coefficient and items with at least 0.70 or higher value were returned.

### Data quality assurance and management

A 4-day training was conducted for research assistants together with supervisors regarding the objectives of the study, data collection techniques, and ethical conduct of research. During training, important information on the availability of HIV service indicators and readiness was emphasized. During data collection, supervisors checked the consistency, completeness of collected data, and facility coverage. On average, data collection took 2 days at each facility, and information regarding availability and readiness for the facility was provided by the ART in-charge. Two investigators (PK and MS) were responsible for daily checks of data submitted online. Detailed data cleaning and validation checks were conducted before analysis.

### Data analysis

We used nine indicators each to measure availability and readiness. A facility was considered to have a given indicator item if it was reported to be present or observed to be available. Aggregates of availability and readiness were calculated from nine indicators for the items present, and the overall proportion of facilities indicating the presence of all items was calculated. Categorical data were described using frequencies and percentages, while continuous variables were described using the median and intertitles ranges. Chi-square tests were used to compare differences between facilities in rural and urban areas.

Since availability and readiness are latent constructs, the relationship with their respective indicators was modeled using the SEM. The model fit was assessed using the chi-square value, root mean square error of approximation (RMSEA), and comparative fit index (CFI). The test level was 0.05 and *p* < 0.05, suggesting significant differences. All statistical analyses were performed using Stata version 17 (Stata Corp., College Station, Texas, United States).

### Ethical considerations

The study was reviewed and approved by the University of Zambia Research Ethics Committee (approval number: 2999-2022), and further permission was obtained from the National Health Research Authority. The study took into consideration procedures to safeguard participants’ confidentiality and privacy. Participants were informed that they were free to withdraw from participating or skip certain questions they felt uncomfortable without any consequences.

## Results

There were 60 public health ART facilities with a median number of clients on ART of 1,225 (interquartile range [IQR], 442-2692). The median number of clients seen per day was 15 (10–20), and the waiting time to be seen was 20 min (10–30). The majority (34, 57.6%) of the facilities were health centers, and 49 (81.7%) had operational hours between 07:00 and 16:00 h. Close to two-thirds 38 (63.3%) were urban facilities and the facilities indicated that 27 (45.0%) of lived more than 5 km from the facilities. Regarding DSD models that were offered by the facilities, all had multi-months dispensing (100%), more than half (6.2%) were offering fast-track, community delivery ART points (46.7%) and only less than one-fifth (15.0%) had family-based model (Table 1).

**Table 1:** Characteristics of selected ART facilities where DSD models were implemented in Zambia, March 2023 (*n* = 60).

Characteristics	Median/Frequency	IQR/Percentage
Clients seen per day in ART, median (IQR)	15	10–20
Waiting time for clients to be seen at ART in minutes, median (IQR)	20	10–30
Number of clients on ART, median (IQR)	1,225	442–2,692
Type of facility		
Level-2 hospital	3	5
Health center	34	57.6
Health post	14	23.7
Level-1 hospital	9	15.3
ART operational times		
07:00–13:00 h	4	6.7
07:00–16:00 h	49	81.7
Anytime	7	11.7
Region		
Rural	22	36.7
Urban	38	63.3
Average distance of clients to the facility (km)		
< 3	6	10
3–5	27	45
> 5	27	45
DSD models offered by facilities*		
Multi-month dispensing	60	100
Fast track	37	61.7
After hour/weekend	26	43.3
Adolescent support	16	26.7
Men’s clinic	13	21.7
Dedicated pediatric ART day	24	40
Community delivery ART points	28	46.7
Scholar model	12	20
Family-based model	9	15
Home ART delivery	17	28.3

ART, antiretroviral; IQR, interquartile range; * multiple response question.

### Availability of HIV service indicators

Facilities with 50% or more availability of HIV service indicators were 81.7% (49/60). The facilities with 50% or more availability of HIV service indicators in rural areas were 74.8%, while for urban facilities, they were 88.7%. Slightly above two-thirds (40, 67.8%) of the facilities reported that they had existing HIV multidisciplinary teams, 56 (94.2%) rolled out the latest ART orientation guidelines, 54 (91.5%) implemented the Zambian HIV quality improvement framework, 53 (88.1%) established a facility-based system for fast-track ART distribution, and almost all facilities (58, 98.3%) had established systems to monitor patient-level HIV treatment outcomes, specifically retention, lost to follow-up, mortalities, and viral load suppression. When different questions about the availability of HIV service indicators were compared between rural and urban facilities, significant differences were found with questions related to the existence of HIV multidisciplinary teams (*p* = 0.002) and the establishment of systems to monitor patient-level outcomes on retention in care, lost to follow-up, mortalities, and viral load suppression (*p* = 0.043), which were more likely to be in urban facilities than in rural facilities (Table 2).

**Table 2:** Availability of basic packages of essential DSD in HIV treatment offered by selected public ART facilities in Zambia, March 2023.

	Questions/Variables	Total *N* = 60	Rural *n* = 22	Urban *n* = 38	*p*-value
Availability, *n* (%) answered Yes					
A1	Does the facility have an existing HIV multidisciplinary team?	40 (67.8)	9 (42.9)	31 (81.6)	0.002
A2	Are the latest ARV orientation guidelines being rolled out at the facility?	56 (94.2)	20 (95.2)	36 (94.7)	0.933
A3	Does the facility have adequate storage space for additional commodities related to HIV services?	43 (72.9)	17 (80.9)	26 (68.4)	0.300
A4	Has the facility implemented the Zambian HIV quality improvement framework?	54 (91.5)	19 (90.5)	35 (92.1)	0.810
A5	Has the facility achieved a routine viral load monitoring uptake of ≥90%?	52 (88.1)	19 (90.5)	33 (86.8)	0.679
A6	Has the facility established a facility-based system for fast-track ART distribution?	53 (89.8)	18 (85.7)	35 (92.1)	0.437
A7	Currently, does the facility have ≥3 months of ART available on site?	56 (94.9)	19 (90.5)	37 (97.4)	0.249
A8	Does the facility have an established system to monitor patient-level outcomes on retention, lost to follow-up, mortalities, and viral load suppression?	58 (98.3)	20 (95.2)	38 (100)	0.175
A9	Is the facility able to establish recording and reporting systems for community ART?	44 (74.6)	18 (85.7)	26 (68.4)	0.144
	**Facilities with all indicators available**			**23/60 (38.3%)**	
	**Facilities with 50% or more indicators**			**49/60 (81.7%)**	

ART, antiretroviral therapy; HIV, human immunodeficiency virus. Bold values are facilities with all indicators available and facilities with at least 50% of the indicators available respectively.

### Facility readiness to provide DSD models

Facility readiness with 50% or more indicators was 81.3% (49/60). The readiness for rural facilities was 73.6%, while for urban facilities, it was 88.8%. The majority (55, 93.2%) of the facilities had community health workers in all departments offering HIV services oriented on the latest ARV guidelines for the year 2022, and quality improvement teams were present in all urban facilities. In addition, HIV multidisciplinary teams that review clinical cases and provide support to patients failing treatment or with advanced disease were more likely to be in urban facilities than in rural facilities (84.2% versus 71.4%). Above three-quarters (47, 79.7%) of the facilities reported that they had identified a focal person to pre-pack and label ART for community distribution, and slightly above half (31, 52.5%) of the facilities had staff and resources to train ART distributors with no difference between rural and urban facilities (Table 3).

**Table 3:** Readiness of selected ART facilities to offer DSD in HIV treatment in Zambia, March 2023.

	Statements/Variables	Total	Rural	Urban	*p*-value
Readiness, *n* (%) answered Yes					
R1	Have community health workers in all departments offering HIV services been oriented on the latest ARV guidelines for the year 2022?	55 (93.2)	19 (90.5)	36 (94.7)	0.048
R2	Does the facility have a commodity management/commodity security committee?	41 (69.5)	15 (71.4)	26 (68.4)	0.810
R3	Does the facility have a quality improvement team?	55 (93.2)	17 (80.9)	38 (100)	0.005
R4	Does the facility have an HIV multidisciplinary team to review clinical cases and provide support to patients failing treatment or with advanced disease?	47 (79.7)	15 (71.4)	32 (84.2)	0.040
R5	At this facility, have the healthcare workers been trained on the revised HIV M&E tools?	43 (72.9)	13 (61.9)	30 (78.9)	0.159
R6	Has the facility identified a focal person to oversee community-based ART distribution?				
R7	Has the facility identified appropriate personnel to distribute ART?	44 (74.6)	17 (80.9)	27 (71.1)	0.403
R8	Does the facility have staff and resources to train ART distributors?	31 (52.5)	12 (57.1)	19 (50.0)	0.599
R9	Has the facility identified a focal person to pre-pack and label ART for community distribution?	47 (79.1)	17 (80.9)	30 (78.9)	0.855
	**Facilities with all readiness indicators**			**29/60 (48.0%)**	
	**Facilities with 50% or more indicators**			**49/60 (81.7%)**	

ART, antiretroviral therapy; HIV, human immunodeficiency; M&E, monitoring and evaluation. Bold values are facilities with all indicators available and facilities with at least 50% of the indicators available respectively.

### Structural equation modeling

**Figure 1:**
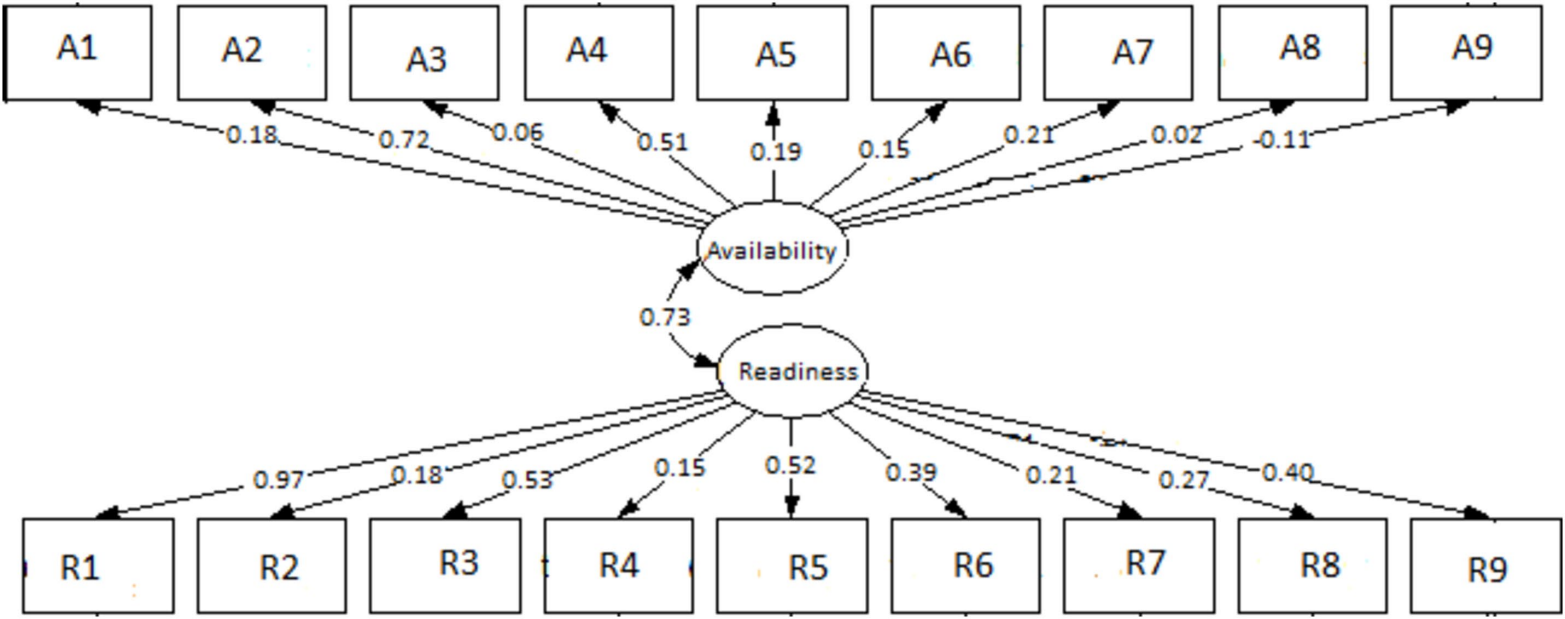
Structure equation modeling (SEM) on the availability and readiness of ART facilities to implement DSD for HIV treatment in selected health facilities in Zambia. A1–A9 are questions on availability, and R1– R9 are questions on readiness.

The SEM was designed to model a relationship between the availability of HIV service indicators and readiness of DSD model provision as well as the moderation effects through the existence and functional structures of ART health facilities. The correlation between availability and readiness was assessed. Taking availability and readiness as latent variables, path analysis of the model showed that the relationship between availability and readiness had a significant positive effect on each other (*r* = 0.73). The presented model suggested an acceptable fit, with RMSEA = 0.012 (<0.08), CFI = 0.96 (>0.95), and an overall chi-square value = 0.09 (>0.05) (18). The coefficients representing the relationships between the variables are indicated by the numbers on the arrows. Direct arrows indicate the direct effect of an explanatory variable on its respective latent variable. There was a significant correlation between the direct effect of the existing HIV multidisciplinary team (r = 0.18), orientation in the latest ART guidelines (r = 0.72), the HIV multidisciplinary team’s role in reviewing clinical cases and supporting patients failing treatment or with advanced disease (r = 0.51), and the identification of appropriate personnel to distribute ART (r = 0.21) with HIV service indicators availability. For readiness, all coefficients were positive and significant. The notable ones were community health workers in all departments offering HIV services having been oriented on the latest ART guidelines for the year 2022 (r = 0.97), the presence of a quality improvement team (r = 0.51), healthcare workers trained in revised HIV monitoring and evaluation tools (r = 0.52), and the identification of a focal person to pre-pack and label ART for community distribution (r = 0.4), as shown in Figure 1.

## Discussion

This study set out to assess the availability of HIV service indicators and readiness to provide DSD models in selected public health facilities in Zambia. The results from the SEM analysis showed that there was a significant correlation between the availability of HIV service indicators and facility readiness to provide DSD models, suggesting that facilities that were considered to have HIV service indicators available were more likely to be ready for the provision of DSD models and vice versa. Availability and readiness of facilities to provide DSD models as defined in this study were above average. There were still gaps in the provision of both indicators for availability and readiness, which requires significant strengthening in equipment, staffing, commodities, and amenities. We believe that a comprehensive assessment of availability and readiness could be maximized by including the qualitative study to show an in-depth understanding of these two constructs that were measured. This is because there is a limitation in the validity of the facility availability and readiness assessment tool (19). Facilities utilized other models apart from the “one-size fits-all,” including the multi-month dispensing model to accelerate the attainment of desired HIV treatment outcomes. The findings continue to highlight essential efforts made in the provision of HIV treatment services to achieve the second and third 95% targets in order to end HIV/AIDS as a public health threat by 2030 (20). DSD model approaches should be adaptive to address specific barriers for all individuals living with HIV to enable them to access treatment easily. Access to treatment is still not equally accessible or used, with some groups encountering specific difficulties. For instance, male individuals with HIV are less likely to access treatment compared to their female counterparts (21). Similarly, adolescents have lower 95-95-95 targets than the general population (22–24). In this regard, specific DSD models for specific groups are essential to assist in closing the gap in the HIV treatment response and can facilitate early access to treatment.

The results of this study showed that the availability of HIV service indicators may culminate in greater provision of DSD models, especially in a more favorable environment due to the observed results from SEM, which showed significant and positive direct standardized effects of some of its indicators. This finding is consistent with another study that demonstrated that the presence of HIV treatment indicators resulted in better provision of treatment (25). However, this is speculative since availability and readiness do not equate to actual implementation of DSD models and desired results, which this study did not assess. In addition, some indicators of availability such as adequate storage space for additional commodities related to HIV services and identification of a focal person to pre-pack and label ART for community distribution were insignificant or negative, suggesting mixed results on their impact on the provision of DSD models. This is not to say they are not important in the provision of the DSD models but could be that on their own or their components may not have a direct association with availability as a latent construct. One plausible explanation could be that each indicator captured a partial aspect that may not be sufficient to represent what the availability of HIV service indicators contains in its entirety. A combination of different important aspects is required for HIV service indicators. For example, although approximately three-quarters of the facilities reported that they had adequate storage space for additional commodities related to HIV services and established recording and reporting systems for community ART, their positive impact may be countered by unfavorable aspects, which could be either missing in the indicators or resulting in the estimated impact of the dimension being insignificant. In this instance, just adequate space for HIV services and having recording and reporting systems for community ART is not sufficient to culminate in the availability of HIV service indicators, especially if the space is not stocked with essential commodities or a non-functional recording and reporting system, respectively.

Determination of a health facility readiness to provide a health service is essential indicator for identification of weaknesses and opportunities for continued improvement (15). This study found that facilities that reported 50% or more of the indicators present were 81.7% suggesting that facilities were ready, and further comparisons showed that urban facilities were more likely to be ready than rural facilities. As suggested by a previous study (26), the observed difference between rural and urban facilities in readiness might be due to a lower supply of resources and essential commodities in rural facilities, which is common in low-resource settings and may contribute to insufficiencies and inequities. Thus, more concerted efforts are needed by all relevant stakeholders to make all indicators of readiness available in rural facilities (27, 28), which serve approximately 60% of Zambia’s population in order to accelerate and increase better HIV treatment outcomes necessary to attain an HIV-free generation and quality care for those living with HIV.

Community health workers oriented in the latest HIV guidelines are important in the provision of HIV treatment services (29, 30). Our study showed that having community health workers oriented in the latest HIV guidelines significantly and positively correlated with the availability of HIV service indicators. In addition, the SEM results showed that the indicator related to the community health workers’ orientation in the latest HIV guidelines had the highest correlation with readiness to implement DSD models. A plausible explanation could be due to the ability of health workers to periodically monitor and provide feedback to improve the quality of services being offered (31), an aspect that may not be possible to implement for health facilities with health workers that have not been oriented in the latest HIV guidelines. Generally, community health workers have been shown to play a pivotal role in the provision of HIV services for better treatment outcomes (32).

Consistent with previous studies (33, 34), this study showed that having a quality improvement team significantly and positively correlated with readiness to provide DSD models. It is possible that facilities with quality improvement teams reviewed and improved signal functions that enhanced DSD models, availability, and readiness. Despite the concerted and innovative efforts in the fight against the HIV pandemic and as a requirement for an HIV framework to improve treatment outcomes, the results showed that all facilities in urban areas but not all in rural areas had quality improvement teams. This could have negatively impacted the readiness and ultimately the ability of facilities in rural areas to effectively provide the DSD models. Ensuring the presence of quality improvement teams in rural facilities may improve service delivery.

This study has several limitations. First, availability and readiness constructs measured may vary significantly depending on the educational, training, and personnel values of individuals who were working in these facilities or interviewed. Second, the degree to which different facilities embraced change, implementation, and adoption of the DSD models for HIV treatment could have varied due to resistance or acceptance at different degrees by staff at each facility. Third, we only used quantitative design to obtain the absence or presence of elements that may influence availability and readiness. This method may make it challenging to accurately measure certain using a binary checklist, potentially leading to underreporting or overreporting of other elements. Future studies should consider including qualitative design to provide a more comprehensive assessment. Moreover, we used the WHO SARA tool, which has been criticized in other studies for reducing the assessment of availability and readiness to a binary checklist. This approach tends to focus heavily on “hardware” systems while neglecting “software” systems, such as interactions among people, values, and norms (35, 36). Therefore, our findings should be interpreted with caution.

In summary, our study suggests some disparities in the availability of HIV service indicators and the readiness of different health facilities to provide the DSD models. Although both availability and readiness were above average, the study highlighted the gaps that exist in certain indicators between rural and urban facilities, such as the availability of quality improvement teams and orientation of community health workers in the latest HIV guidelines. With respect to readiness, differences were noted in the area of existence of HIV multidisciplinary teams to review clinical cases and provide support to patients who are failing treatment or those with advanced disease. This calls for training and the provision of guidelines to strengthen HIV service necessary for HIV treatment and care for better treatment outcomes. Therefore, the stakeholders and the government should prioritize the training and orientation of community health workers, the establishment of quality improvement teams, and the setting up of multidisciplinary teams to review HIV clinical cases. Future studies should consider longitudinal or panel data collection to strengthen the non-experimental approach to causal analysis (37) and may be useful to ensure the effective implementation of signal functions of the availability and readiness of health facilities to provide HIV DSD models. The evidence in this study could inform onward planning with respect to strengthening areas that are negatively affecting the effective provision of DSD of HIV treatment. Indicators that were positive and significant in SEM would potentially improve DSD models and probably better HIV treatment outcomes.

## Data Availability

The raw data supporting the conclusions of this article will be made available by the authors, without undue reservation.

## References

[1] Ministry of Health of Zambia: Zambia Differentiated service delivery (DSD) framework (2022–2026).

[2] Huber A Pascoe S Nichols B Long L Kuchukhidze S Phiri B. Differentiated service delivery models for HIV treatment in Malawi, South Africa, and Zambia: a landscape analysis. Glob Health Sci Pract. (2021) 9:296–307. doi: 10.9745/GHSP-D-20-00532, PMID: 34234023 PMC8324204

[3] Long L Kuchukhidze S Pascoe S Nichols B Cele R Govathson C. Differentiated models of service delivery for antiretroviral treatment of HIV in sub-Saharan Africa: a rapid review protocol. Syst Rev. (2019) 8:314. doi: 10.1186/s13643-019-1210-6, PMID: 31810482 PMC6896778

[4] Luque-Fernandez MA Van Cutsem G Goemaere E Hilderbrand K Schomaker M Mantangana N. Effectiveness of patient adherence groups as a model of care for stable patients on antiretroviral therapy in Khayelitsha, Cape Town, South Africa. PLoS One. (2013) 8:e56088. doi: 10.1371/journal.pone.0056088, PMID: 23418518 PMC3571960

[5] Grimsrud A Bygrave H Doherty M Ehrenkranz P Ellman T Ferris R. Reimagining HIV service delivery: the role of differentiated care from prevention to suppression. J Int AIDS Soc. (2016) 19:21484. doi: 10.7448/IAS.19.1.21484, PMID: 27914186 PMC5136137

[6] Wouters E Van Damme W van Rensburg D Masquillier C Meulemans H. Impact of community-based support services on antiretroviral treatment programme delivery and outcomes in resource-limited countries: a synthetic review. BMC Health Serv Res. (2012) 12:194. doi: 10.1186/1472-6963-12-194, PMID: 22776682 PMC3476429

[7] Murray KR Dulli LS Ridgeway K Dal Santo L. Darrow de Mora D, Olsen P, Silverstein H, McCarraher DR: improving retention in HIV care among adolescents and adults in low-and middle-income countries: a systematic review of the literature. PLoS One. (2017) 12:e0184879. doi: 10.1371/journal.pone.0184879, PMID: 28961253 PMC5621671

[8] World Health Organisation: Consolidated guidelines on the use of antiretroviral drugs for treating and preventing HIV infection: recommendations for a public health approach 2016 (2nd edition). 27466667

[9] Kruk ME Gage AD Arsenault C Jordan K Leslie HH Roder-DeWan S. High-quality health systems in the sustainable development goals era: time for a revolution. Lancet Glob Health. (2018) 6:e1196–252. doi: 10.1016/S2214-109X(18)30386-3, PMID: 30196093 PMC7734391

[10] Abdella A Fetters T Benson J Pearson E Gebrehiwot Y Andersen K. Meeting the need for safe abortion care in Ethiopia: results of a national assessment in 2008. Glob Public Health. (2013) 8:417–34. doi: 10.1080/17441692.2013.778310, PMID: 23590804

[11] Zambia Statistical Agency: Zambia’s Total population by province. (2022). Availablea at: https://www.zamstats.gov.zm (Accessed February 23, 2024).

[12] Ministry of Health, Zambia. Zambia Population-based HIV Impact Assessment (ZAMPHIA) 2021: Final Report. Lusaka, Ministry of Health. Available at: http://phia.icap.columbia.edu (Accessed February 27, 2024).

[13] Liu C Liu C Wang D Deng Z Tang Y Zhang X. Determinants of antibiotic prescribing behaviors of primary care physicians in Hubei of China: a structural equation model based on the theory of planned behavior. Antimicrob Resist Infect Control. (2019) 8:23. doi: 10.1186/s13756-019-0478-6, PMID: 30733857 PMC6354420

[14] Namasivayam A Arcos González P Castro Delgado R Chi PC. The effect of armed conflict on the utilization of maternal health Services in Uganda: a population-based study. PLoS Curr. (2017) 9:ecurrents.dis.557b987d6519d8c7c96f2006ed3c271a. doi: 10.1371/currents.dis.557b987d6519d8c7c96f2006ed3c271a 29188138 PMC5693797

[15] World Health Organisation: Service availability and readiness assessment (SARA) tool. An annual monitoring system for service delivery. Reference manual (2015), version 2.2. (Accessed February 28, 2024).

[16] Mahipala PG Afzal S Uzma Q Aabroo A Hemachandra N Footman K. An assessment of facility readiness for comprehensive abortion care in 12 districts of Pakistan using the WHO Service availability and readiness assessment tool. Sex Reprod Health Matters. (2023) 31:2178265. doi: 10.1080/26410397.2023.2178265, PMID: 36897212 PMC10013260

[17] Kobo Collect/Toolbox. In: Powerful and intuitive data collection tools to make an impact. (2023). Available at: https://www.kobotoolbox.org/

[18] Hu LT Bentler PM. Cutoff criteria for fit indexes in covariance structure analysis: conventional criteria versus new alternatives. Struct Equ Model Multidiscip J. (1999) 6:1–55. doi: 10.1080/10705519909540118

[19] Namutebi M Nalwadda GK Kasasa S Muwanguzi PA Ndikuno CK Kaye DK. Readiness of rural health facilities to provide immediate postpartum care in Uganda. BMC Health Serv Res. (2023) 23:22. doi: 10.1186/s12913-023-09031-4 36627623 PMC9830711

[20] Frescura L Godfrey-Faussett P Feizzadeh AA El-Sadr W Syarif O Ghys PD. On, behalf of the testing treatment target working G: achieving the 95 95 95 targets for all: a pathway to ending AIDS. PLoS One. (2022) 17:e0272405. doi: 10.1371/journal.pone.0272405, PMID: 35925943 PMC9352102

[21] Castilho JL Melekhin VV Sterling TR. Sex differences in HIV outcomes in the highly active antiretroviral therapy era: a systematic review. AIDS Res Hum Retrovir. (2014) 30:446–56. doi: 10.1089/aid.2013.0208, PMID: 24401107 PMC4010172

[22] Reif LK Abrams EJ Arpadi S Elul B McNairy ML Fitzgerald DW. Interventions to improve antiretroviral therapy adherence among adolescents and youth in low-and middle-income countries: a systematic review 2015–2019. AIDS Behav. (2020) 24:2797–810. doi: 10.1007/s10461-020-02822-4, PMID: 32152815 PMC7223708

[23] Salou M Dagnra AY Butel C Vidal N Serrano L Takassi E. High rates of virological failure and drug resistance in perinatally HIV-1-infected children and adolescents receiving lifelong antiretroviral therapy in routine clinics in Togo. J Int AIDS Soc. (2016) 19:20683. doi: 10.7448/IAS.19.1.20683, PMID: 27125320 PMC4850147

[24] Adejumo OA Malee KM Ryscavage P Hunter SJ Taiwo BO. Contemporary issues on the epidemiology and antiretroviral adherence of HIV-infected adolescents in sub-Saharan Africa: a narrative review. J Int AIDS Soc. (2015) 18:20049. doi: 10.7448/IAS.18.1.20049, PMID: 26385853 PMC4575412

[25] Loncar D Izazola-Licea JA Krishnakumar J. Exploring relationships between HIV programme outcomes and the societal enabling environment: a structural equation modeling statistical analysis in 138 low-and middle-income countries. PLOS Glob Public Health. (2023) 3:e0001864. doi: 10.1371/journal.pgph.0001864, PMID: 37159438 PMC10168546

[26] Hakim S Chowdhury MAB Haque MA Ahmed NU Paul GK Uddin MJ. The availability of essential medicines for cardiovascular diseases at healthcare facilities in low-and middle-income countries: the case of Bangladesh. PLOS Glob Public Health. (2022) 2:e0001154. doi: 10.1371/journal.pgph.0001154, PMID: 36962886 PMC10021517

[27] Spasojevic N Vasilj I Hrabac B Celik D. Rural-urban differences in health care quality assessment. Mater Sociomed. (2015) 27:409–11. doi: 10.5455/msm.2015.27.409-411, PMID: 26937222 PMC4753384

[28] Oyekale AS. Assessment of primary health care facilities' service readiness in Nigeria. BMC Health Serv Res. (2017) 17:172. doi: 10.1186/s12913-017-2112-8, PMID: 28249578 PMC5333428

[29] Ngcobo S Scheepers S Mbatha N Grobler E Rossouw T. Roles, barriers, and recommendations for community health workers providing community-based HIV Care in sub-Saharan Africa: a review. AIDS Patient Care STDs. (2022) 36:130–44. doi: 10.1089/apc.2022.0020, PMID: 35438523 PMC9057893

[30] Busza J Dauya E Bandason T Simms V Chikwari CD Makamba M. The role of community health workers in improving HIV treatment outcomes in children: lessons learned from the ZENITH trial in Zimbabwe. Health Policy Plan. (2018) 33:328–34. doi: 10.1093/heapol/czx187, PMID: 29309578 PMC5886269

[31] Muwonge TR Nsubuga R Ware NC Wyatt MA Pisarski E Kamusiime B. Health care worker perspectives of HIV pre-exposure prophylaxis service delivery in Central Uganda. Front Public Health. (2022) 10:658826. doi: 10.3389/fpubh.2022.658826, PMID: 35444979 PMC9013815

[32] Naidoo N Matlakala N Railton J Khosa S Marincowitz G Igumbor JO. Provision of HIV services by community health workers should be strengthened to achieve full programme potential: a cross-sectional analysis in rural South Africa. Trop Med Int Health. (2019) 24:401–8. doi: 10.1111/tmi.13204, PMID: 30637860 PMC6445684

[33] Gaga S Mqoqi N Chimatira R Moko S Igumbor JO. Continuous quality improvement in HIV and TB services at selected healthcare facilities in South Africa. South Afr J HIV Med. (2021) 22:1202. doi: 10.4102/sajhivmed.v22i1.1202, PMID: 34192068 PMC8182456

[34] Ikeda DJ Nyblade L Srithanaviboonchai K Agins BD. A quality improvement approach to the reduction of HIV-related stigma and discrimination in healthcare settings. BMJ Glob Health. (2019) 4:e001587. doi: 10.1136/bmjgh-2019-001587, PMID: 31297246 PMC6590995

[35] Arakelyan S Mac Gregor H Voce AS Seeley J Grant AD Kielmann K. Beyond checklists: using clinic ethnography to assess the enabling environment for tuberculosis infection prevention control in South Africa. PLOS Glob Public Health. (2022) 2:e0000964. doi: 10.1371/journal.pgph.0000964, PMID: 36962641 PMC10022266

[36] Kielmann K Dickson-Hall L Jassat W Le Roux S Moshabela M Cox H. “We had to manage what we had on hand, in whatever way we could”: adaptive responses in policy for decentralized drug-resistant tuberculosis care in South Africa. Health Policy Plan. (2021) 36:249–59. doi: 10.1093/heapol/czaa147, PMID: 33582787 PMC8059133

[37] Wegener DT Fabrigar LR. Analysis and desgin for nonexperimental data: addressing causal and noncausal hypothesis. In: HT Reis CM Judd, editors. Handbook of research methods in social and personality psychology. edn ed. New York, NY, US: Cambridge University Press (2000). 412–50.

